# Neural network surrogates enable robust and accessible modeling of renal epithelial transport

**DOI:** 10.14814/phy2.70921

**Published:** 2026-05-18

**Authors:** Anita T. Layton

**Affiliations:** ^1^ Department of Applied Mathematics and Biology, Cheriton School of Computer Science, School of Pharmacy University of Waterloo Waterloo Ontario Canada

**Keywords:** artificial intelligence, epithelial transport, kidney, machine learning

## Abstract

Mathematical models of renal epithelial transport provide powerful tools for investigating kidney function, but their broader use is often hindered by numerical challenges. These highly nonlinear model equations typically require specialized solvers and careful initialization, which can reduce robustness and accessibility. To address these limitations, we propose a neural‐network (NN)–based framework for approximating steady‐state solutions of epithelial transport models. Training and validation data are generated using published mechanistic models by systematically varying transport parameters and computing corresponding cellular states, including solute concentrations, membrane potentials, and fluxes. The NN is then trained as a fast surrogate for the mechanistic model. Once trained, the NN accurately predicts cellular variables for previously unseen parameter sets. Using proximal convoluted tubule and medullary thick ascending limb cells as examples, we demonstrate high predictive accuracy when training and testing data are drawn from the same sex. Cross‐sex predictions are less accurate, reflecting known sex differences in transporter abundance. The NN also accurately models transporter inhibition scenarios and can be trained to infer membrane transport properties from measurable physiological data, highlighting its potential for inverse modeling. Overall, this NN‐based solver provides a robust alternative to traditional approaches, eliminating convergence failures and improving accessibility of epithelial transport models.

## Graphical abstract

This study introduces a neural network–based surrogate that replaces fragile numerical solvers in mechanistic models of renal epithelial transport. By learning steady‐state solutions from high‐fidelity simulations, the approach delivers fast, robust predictions across physiological conditions, including transporter inhibition, while avoiding convergence failures. The work further demonstrates inverse inference of transport properties from measurable data and reveals sex‐specific limits to generalization, opening new avenues for accessible, scalable, and physiologically grounded kidney modeling.

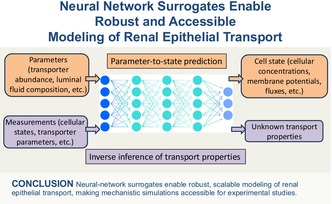

## 1 INTRODUCTION

Renal tubular transport is vital for maintaining homeostasis by adjusting water and solute reabsorption along the nephrons such that excretion matches daily intake. This regulation is mediated by hormonal and neuronal signals and depends on the functional diversity of tubular segments. The nephron comprises sequential segments: from the proximal tubule, loop of Henle, distal convoluted tubule, connecting tubule, to the collecting duct, each lined by specialized epithelial cells with distinct transport roles. The proximal tubule reabsorbs the bulk of filtered water, NaCl, and nutrients. The thick ascending limb of the loop of Henle pumps NaCl without water, diluting the tubular fluid. The collecting duct fine‐tunes water reabsorption depending on the body's hydration status, helping to concentrate or dilute urine (Eaton, 2009; Layton & Sands, 2026).

To investigate physiological processes and function changes of the kidney in diseases, one may employ a useful and noninvasive approach: computational modeling (Layton & Edwards, 2014). Detailed models of solute and fluid transport have been developed for renal epithelial cells (Layton, 2024a; Wei et al., 2018; Weinstein, 1983), tubular segments (Hakimi et al., 2023; Layton & Layton, 2018; Stadt & Layton, 2022; Weinstein, 2015), and populations of nephrons (Hu et al., 2021; Hu & Layton, 2021; Layton et al., 2018). Formulating a mathematical model of renal epithelial cellular transport involves describing how solutes and water move across the different compartments of an epithelial cell, typically including the luminal (apical) space, the cytoplasm, and the basolateral (interstitial) space. These transport processes are mediated by a variety of channels, pumps, and transporters, each governed by biophysical laws such as the Goldman–Hodgkin–Katz equation, Michaelis–Menten kinetics, or electro‐diffusive flux equations (Layton & Edwards, 2014). The model's state variables include solute concentrations in different compartments (lumen, cytosol, lateral innerspace (LIS), and interstitium), membrane potentials, cell volume, and sometimes buffering agents. At its core, the model is built on mass balance equations that describe the rates of change of solute concentrations, coupled with algebraic constraints such as electroneutrality. Because of these algebraic relationships and the nonlinear kinetics of transport processes, the model forms a system of coupled nonlinear differential‐algebraic equations. These equations are strongly interdependent: for example, a change in sodium concentration affects membrane potential, which alters potassium flux, influencing osmolarity and water movement, thereby affecting cell volume and additional transport pathways. The system typically includes a large number of parameters, such as membrane permeabilities, transporter kinetics (e.g., Vmax and Km), surface areas, and stoichiometries, which must be estimated from experimental data. This modeling approach enables researchers to explore how epithelial cells regulate solute and water transport under normal and pathophysiological conditions and to simulate the effects of pharmacological interventions or genetic defects on renal function.

Computing the solution to these models requires specialized numerical techniques for stiff systems of differential algebraic equations, as well as careful initialization to satisfy both differential and algebraic components (Butcher, 2016; Layton & Edwards, 2014; Layton & Layton, 2002). Nonlinear solvers are typically employed; however, because of the stiffness of the system, convergence is not guaranteed unless the initial guess lies sufficiently close to the true solution. Although these challenges are not insurmountable, they demand numerical expertise and have likely limited the broader adoption of epithelial transport models by non‐computational scientists. In practice, while publicly available models may run under baseline conditions, modifying parameters to explore alternative physiological or pathological scenarios often leads to solver failure. Motivated by these limitations, the present study proposes a more robust and user‐friendly approach for approximating steady‐state solutions to epithelial transport models using machine learning.

Recent applications of machine learning in renal physiology and pathophysiology have focused primarily on tasks such as segmenting kidney structures from imaging data and predicting disease states, including acute kidney injury and chronic kidney disease, from electronic health records (Layton, 2023, 2024b, 2025). In contrast, the present study demonstrates how machine learning can be integrated with mechanistic modeling to advance investigations of renal epithelial transport. Rather than developing a new mechanistic model, our objective is to introduce a robust neural‐network (NN)–based surrogate for existing epithelial transport models that approximates their steady‐state solutions while avoiding the numerical failures commonly encountered with traditional solvers. Published transport models are typically calibrated for a specific set of transport parameters and hemodynamic conditions; the proposed surrogate extends their practical utility by enabling reliable exploration of alternative physiological scenarios through parameter variation. In addition, the surrogate can be used to infer membrane transport properties from measurable physiological data, highlighting its potential for inverse modeling. Below, we present several proof‐of‐concept examples to illustrate the versatility and utility of this approach.

## 2 METHODS

### 2.1 Mathematical model for renal epithelial transport

We first describe the mathematical model whose steady‐state solutions are approximated by the neural network solver. The model represents solute and fluid exchange between a renal epithelial cell and its surrounding fluid compartments. By specifying appropriate membrane transport properties, the framework can represent different renal epithelial cell types, including proximal tubule, thick ascending limb, distal convoluted tubule, connecting tubule, and collecting duct cells. In addition, by adjusting parameter values, the model can simulate epithelial cells from male or female rat kidneys. The models differ in tubular dimensions and in the expression levels of selected membrane transporters and channels, reflecting experimentally observed sex differences in renal transport physiology. These differences are implemented as parameter variations (e.g., transporter activity and permeability coefficients) within a common model structure. A detailed account of the sex‐specific parameter values and their physiological basis can be found in previous work (Layton, 2022).

The model consists of four compartments, the lumen, the cell, the LIS, and the interstitium. Fifteen solutes are represented: Na^+^, K^+^, Cl^−^, HCO_3_^−^, H_2_CO_3_, CO_2_, NH_3_, NH_4_^+^, HPO_4_^2−^, H_2_PO_4_^−^, H^+^, HCO_2_^−^, H_2_CO_2_, urea, and glucose. The composition of the lumen and interstitial fluid is assumed known a priori. Water and solutes can be exchanged (reabsorbed or secreted) between the lumen and interstitium via two pathways: the transcellular route across the cell, or the paracellular route through the LIS.

The model consists of a large system of coupled differential algebraic equations. Model equations describe mass conservation and electroneutrality in the cellular and paracellular compartments, and can be found in Ref (Layton, 2022). Model solution predicts cytosolic and LIS solute concentrations, cell volume, epithelial membrane potentials, and transcellular and paracellular fluxes.

The model is typically solved at steady state using a nonlinear solver, most commonly Newton's method or one of its variants. As noted above, successful convergence of a nonlinear solver requires an initial guess that lies sufficiently close to the true solution. In renal epithelial transport models, however, this radius of convergence is often small. Consequently, an initial guess that converges for a published parameter set may fall outside the basin of attraction when transport parameters are modified to represent alternative physiological or pathological conditions, such as transporter inhibition, leading to solver failure. These limitations motivate the present study, which seeks to develop a robust numerical approach that avoids such convergence issues and enables reliable exploration of epithelial transport behavior across a broader range of conditions.

### 2.2 A neural network‐based framework

We propose a NN–based surrogate for mathematical models of renal epithelial transport. NNs are machine learning models inspired by biological neural systems and consist of layers of interconnected nodes that transform input data through weighted connections to learn complex input–output relationships. In this framework, the NN serves two complementary purposes. First, it approximates the steady‐state solution of an epithelial transport model: given a set of cellular transport parameters, it predicts cellular composition, cell volume, membrane potentials, and transmembrane and paracellular fluxes. Second, the NN can be used in an inverse setting to infer underlying transport properties from measurable physiological data. In both cases, the surrogate mitigates the numerical convergence challenges commonly encountered with traditional nonlinear solvers.

Training the NN requires a dataset consisting of many examples of transport parameters paired with their corresponding cellular states. During this training phase, the NN learns the underlying relationships between transporter activities, membrane permeabilities, and steady‐state cellular variables. Once trained, the NN can generalize this learned mapping to previously unseen parameter sets, enabling rapid prediction without explicit numerical integration. For example, the surrogate can be used to estimate how a reduction in basolateral Na^+^/K^+^‐ATPase activity would alter cytosolic K^+^ concentration or basolateral membrane potential. In this way, the NN provides an efficient and robust tool for exploring epithelial transport behavior under diverse physiological and pathological conditions.

As a representative example, we consider epithelial cells from the proximal convoluted tubule (PCT) and the medullary thick ascending limb (mTAL) of the rat kidney. NNs are trained for each cell type and subsequently used to generate predictions. Training data are produced using published mathematical models of epithelial transport along a superficial rat nephron (hereafter referred to as the mechanistic models) (Dutta & Layton, 2024). Although the present study focuses on the PCT and mTAL, the same framework can be readily extended to other renal epithelial cell types, including the distal convoluted tubule, connecting tubule, and collecting duct. To construct the training dataset, selected baseline transport parameters for the PCT and mTAL are systematically varied over physiologically relevant ranges (50%–200% of baseline value), and the mechanistic models are used to compute the corresponding steady‐state cellular variables. To ensure robust convergence of the underlying nonlinear solver, parameter values are varied progressively, with each simulation initialized using the solution from a nearby parameter set. This continuation‐type approach reduces the likelihood of convergence failure when exploring the parameter space.

For this proof‐of‐concept study, we restrict attention to a limited set of features and outputs, with four input parameters and four output variables. We employ a relatively simple NN architecture consisting of two hidden layers, each with 16 nodes, which we found to provide satisfactory accuracy for the selected inputs and outputs. More complex architectures with additional layers or nodes would be required to accommodate higher‐dimensional feature sets. Rectified linear units (ReLU) are used as the activation function, along with a dropout rate of 0.1 to mitigate overfitting. Model performance is evaluated using an 80:20 training–test split.

## 3 RESULTS

### 3.1 Predicting cellular concentrations and membrane potentials

#### 3.1.1 Proximal convoluted tubule (PCT)

Two NNs are trained using rat data, one for male and one for female. Input features are Na^+^‐H^+^ exchanger 3 (NHE3) activity, Na^+^/K^+^‐ATPase activity, Na^+^‐HCO_3_^−^ cotransporter (NBCe1) activity, and basolateral K^+^ permeability. Output variables are the cytosolic [Na^+^] and [K^+^], and apical and basolateral membrane potentials (V_a_ and V_b_). The selected parameters and variables are intended as a proof of concept. The NN is not limited to these specific choices or to investigations involving only Na^+^ and K^+^; additional solute concentrations and transport parameters can be incorporated as needed. Each dataset consists of 4096 samples, which, while modest in size, are sufficient for the selected number of input features and output variables, as demonstrated by the performance results presented below. Expanding the number of features or outputs would require larger datasets and/or more complex network architectures with additional layers or nodes.

**FIGURE 1:**
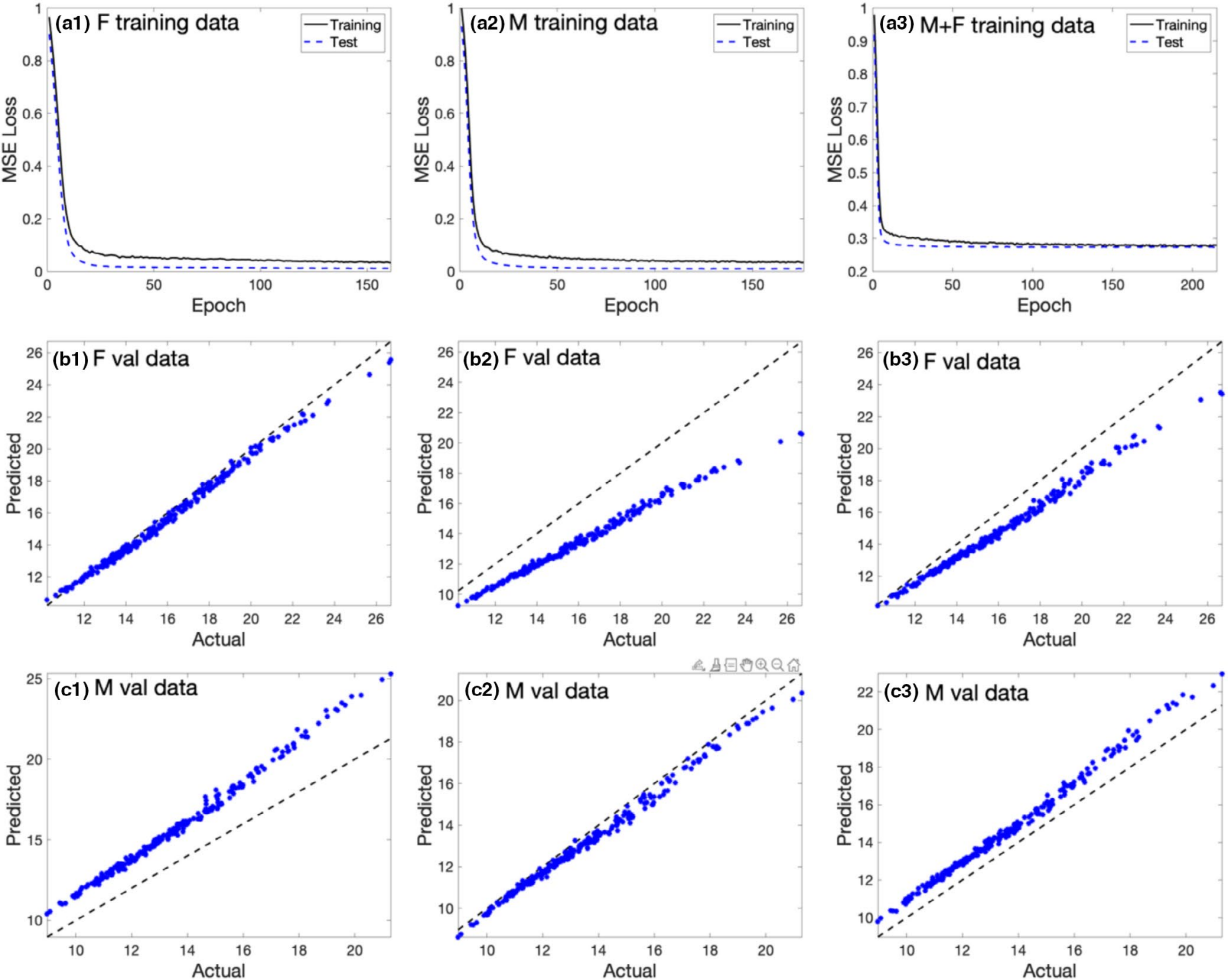
Row a, errors of model predictions on the training and test datasets decrease as training proceeds. a1, a2, and a3 show results for neural networks (NNs) trained using data from female (F), male (M), and both sexes combined (M + F), respectively. All three models exhibit rapid convergence. Rows b and c, validation plots showing predicted cytosolic [Na^+^] versus actual values. Dashed lines correspond to perfect accuracy with predicted equal actual values. Row b, female validation data; row c, male validation data. For both single‐sex models, cross‐sex validation yields low accuracy (c1, b2). Sex‐inclusive models improve accuracy for both sexes (b3, c3).

As shown in Figure 1, a1–a3, both training and test errors decrease as training progresses. Each plot displays the value of the loss function—defined as the difference between predicted variables and their corresponding true values—as a function of epoch, where one epoch corresponds to a single pass through the entire training dataset. After training, the NNs are evaluated on a validation set consisting of 250 previously unseen data points, with transporter parameters randomly sampled within physiological ranges. The resulting prediction errors are summarized in Table 1 (rows labeled “PCT (M)” and “PCT (F)”). Errors for one representative output variable, cytosolic Na^+^ concentration, are shown in Figure 1 (rows b and c). When training and validation data are drawn from the same sex (Figure 1, b1 and c2), both networks achieve high predictive accuracy. In contrast, cross‐sex predictions exhibit substantially larger errors (Figure 1, b2 and c1), consistent with known sex differences in renal transport properties reported in rodent kidneys (Hu et al., 2021).

**TABLE 1:** Fractional mean square errors (MSE) in model predictions, obtained for the proximal convoluted tubule (PCT) and medullary thick ascending limb (mTAL).

Cell type	Validation	MSE (%)	Validation	MSE (%)
PCT (M)	M	3.3	F	13.5
PCT (F)	F	4.3	M	23.0
PCT (M + F)	M	12.0	F	6.7
mTAL (M)	M	2.5	F	27.2
mTAL (F)	F	1.8	M	20.2
mTAL (M + F)	M	2.2	F	3.0

*Note*: For each cell type, three neural network models are generated, trained separately on data from male (M), female (F), and both (M + F), as indicated under Cell type. Each model is tested against M and F validation datasets.

#### 3.1.2 Medullary thick ascending limb (mTAL)

Analogous computations are done for the mTAL. As in the PCT case, two NNs are trained using rat data, one for male and one for female. The input features for the mTAL model include Na^+^‐K^+^‐Cl^−^ cotransporter 2 (NKCC2) activity, Na^+^/K^+^‐ATPase activity, apical K^+^ permeability, and basolateral K^+^ permeability. Validation results are qualitatively similar to the PCT; see Table 1, rows labeled “mTAL (M)” and “mTAL (F)”.

#### 3.1.3 Sex‐inclusive models

We next examine whether a model trained on both male and female rat data can accurately predict cellular states for each sex. To this end, we train sex‐inclusive NNs for the PCT and mTAL. Overall, these models yield improved predictive accuracy for both sexes. In the mTAL, the sex‐inclusive model achieves accuracy comparable to that of the corresponding sex‐specific models. In contrast, although the sex‐inclusive model improves prediction accuracy for the PCT relative to cross‐sex models, its performance remains lower than that of sex‐specific models, as expected (see Table 1 and Figure 1, panels b3 and c3). The stronger performance of the sex‐inclusive model in the mTAL likely reflects the inclusion of key transporters exhibiting sex‐dependent expression—namely NKCC2 and Na^+^/K^+^‐ATPase—within the feature set. By comparison, the PCT feature set does not include aquaporin 1 (AQP1) or claudin 2, whose abundances are known to exhibit significant sex differences, potentially limiting the model's ability to fully capture sex‐specific transport behavior (Hu et al., 2019).

### 3.2 Predicting cellular state under transporter inhibition

**FIGURE 2:**
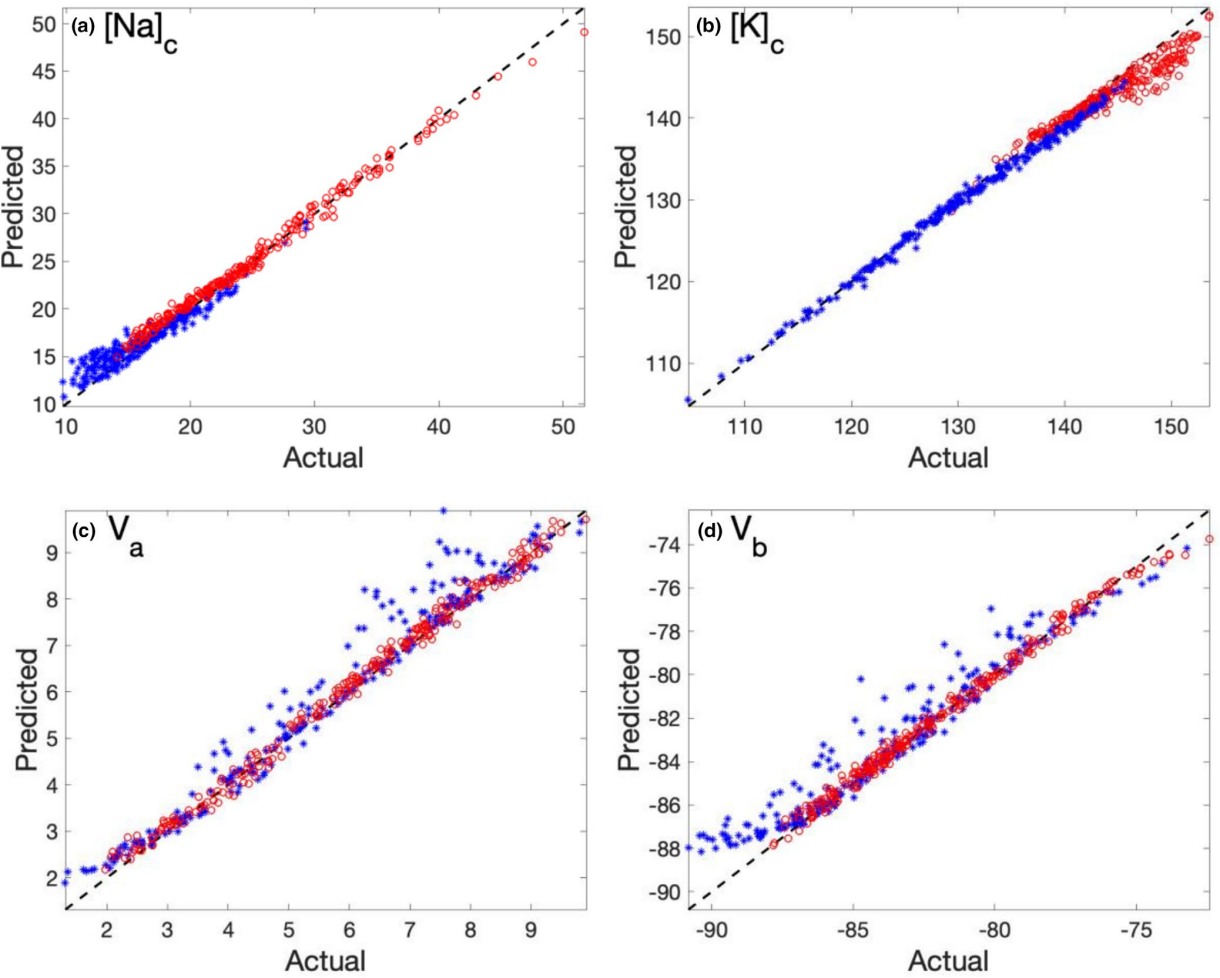
Scattered plots of predicted variables versus actual values, obtained for the female mTAL model. Red dots, baseline transporter parameters; blue dots, NKCC2 is 70% inhibited. Dashed lines correspond to perfect accuracy with predicted equal actual values. (a) cytosolic [Na^+^]; (b) cytosolic [K^+^]; (c) apical membrane potential V_a_; (d) basolateral membrane potential V_b_.

We next evaluate the ability of the NNs to predict the effects of transporter inhibition. In these simulations, the expression level of one or more transporters is sampled from a distribution that lies outside (specifically, below) the range used for network training. For the PCT, we consider inhibition of the Na^+^/H^+^ exchanger NHE3, with the mean expression level reduced by 40% relative to the baseline distribution. For the mTAL, we simulate a 70% reduction of the baseline NKCC2 activity distribution. Other parameters are kept at their baseline distributions. Prediction errors, quantified by the mean squared error (MSE), are summarized in Table 2. For most output variables, errors remain below 10%, with the exception of the apical membrane potential (V_a_). The heightened sensitivity of V_a_ to changes in NHE3 and NKCC2 suggests that these transporters play a dominant role in determining the apical membrane potential. Figure 2 presents scatter plots comparing predicted and actual values of cytosolic Na^+^ and K^+^ concentrations, as well as apical and basolateral membrane potentials, for the female mTAL. Results for the inhibition and baseline cases are shown in blue and red, respectively. As expected, prediction errors are larger under inhibition than under baseline conditions, reflecting the fact that the inhibition scenarios involve transporter expression levels that are largely outside the range represented in the training data.

**TABLE 2:** Fractional mean square errors (MSE) in model predictions, obtained for the proximal convoluted tubule (PCT) with 40% NHE3 inhibition, and for the medullary thick ascending limb (mTAL) with 70% NKCC2 inhibition.

Cell type	Sex	[Na^+^]	[K^+^]	V_a_	V_b_
PCT	M	4.3	0.5	20	1.0
PCT	F	3.7	0.4	29	0.9
mTAL	M	10	4,0	18	1.3
mTAL	F	6.7	2.3	8.0	1.4

Abbreviations: V_a_, apical membrane potential; V_b_, basolateral membrane potential.

### 3.3 Predicting membrane transport properties: The inverse problem

In the final set of experiments, we evaluate the ability of the NNs to infer membrane transport properties from measurable physiological data. To this end, the networks are retrained using data generated from the same male and female rat PCT models, but with the input features defined as observable quantities: cytosolic Na^+^ and K^+^ concentrations, along with transcellular Na^+^ and K^+^ fluxes. The NNs are then tasked with predicting NHE3 activity, Na^+^/K^+^‐ATPase activity, NBCe1 activity, and basolateral K^+^ permeability. The choice of input variables in the inverse problem is not unique and is guided by the availability and informativeness of measurable quantities. In this study, we select cytosolic Na^+^ and K^+^ concentrations and transcellular Na^+^ and K^+^ fluxes as representative observables that are closely coupled to key transport processes. These variables provide strong constraints on transporters that directly regulate Na^+^ and K^+^ balance, such as Na^+^/K^+^‐ATPase and K^+^ channels. More generally, the inverse framework can incorporate different combinations of measurable variables (e.g., membrane potentials, fluxes, or solute concentrations), and the success of parameter inference depends on how strongly those variables reflect the underlying transport mechanisms.

**FIGURE 3:**
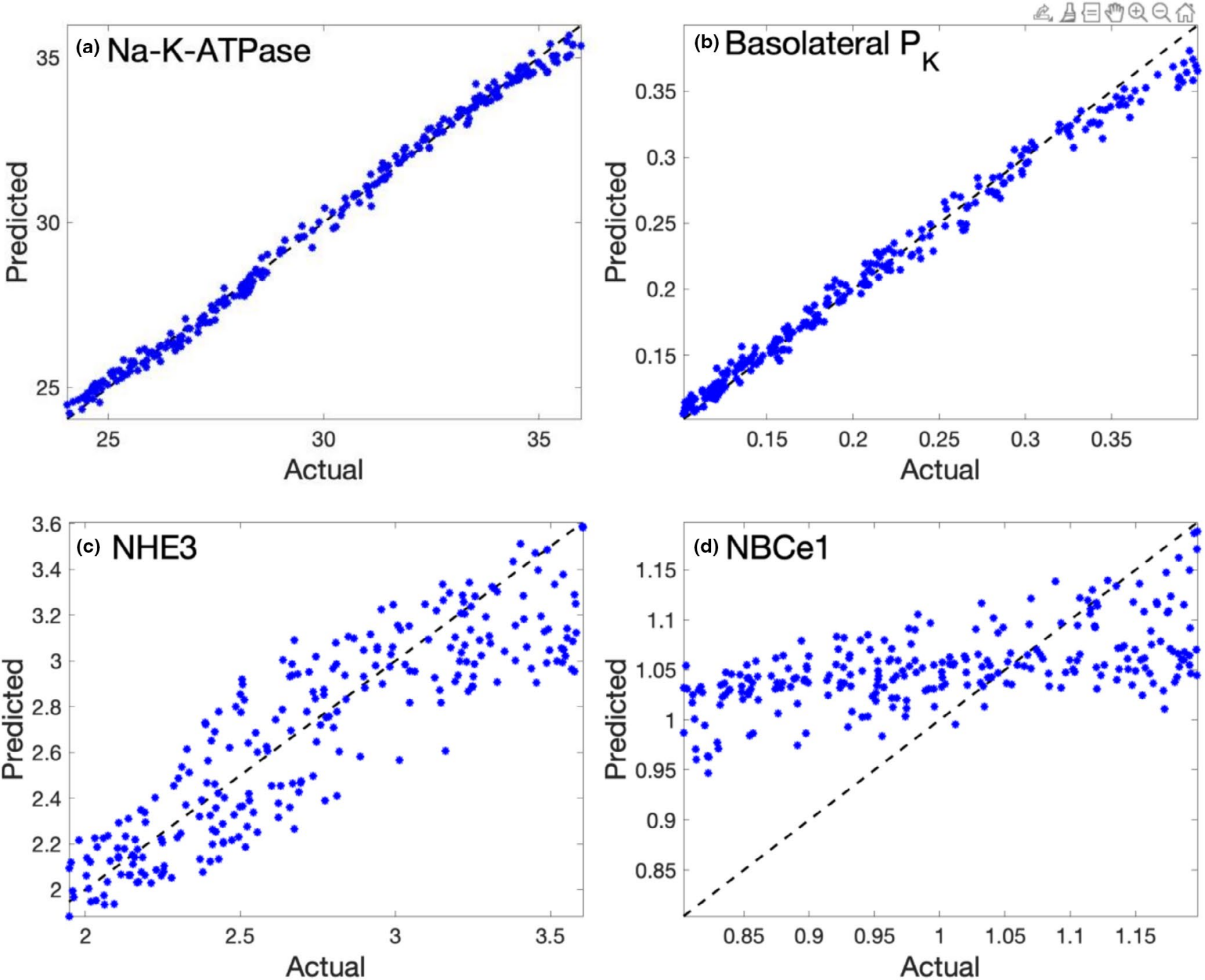
Scattered plots of predicted variables versus actual values, obtained for the male proximal convoluted tubule model. Dashed lines correspond to perfect accuracy with predicted equal actual values. Predictions of Na^+^/K^+^‐ATPase and basolateral P_K_ (a and b) are accurate, whereas errors are larger for NHE3 (c), and NBCe1 is essentially unrecoverable (d).

Prediction errors are summarized in Table 3 and illustrated in Figure 3. The results indicate that the networks accurately recover Na^+^/K^+^‐ATPase activity, reflecting its dominant role in governing Na^+^ and K^+^ fluxes. Basolateral K^+^ permeability is also well identified, owing to its close relationship with K^+^ transport. In contrast, while the networks capture the overall trend in NHE3 activity, they do not reliably recover its precise value, likely because NHE3 competes with other apical Na^+^ transport pathways, most notably the Na^+^–glucose cotransporter 2 (SGLT2).

**TABLE 3:** Mean squared errors (MSE) in model predictions (selected transporter parameters) for different cell types, sex, and transporter inhibition status.

Cell type	Sex	NaKATPase	Basolateral P_K_	NHE3	NBCe1
PCT	M	1.5	5.2	8.2	11.6
PCT	F	1.5	5.4	13.1	21.2

		NKCC2	NaKATPase	Apical P_K_	Basolateral P_K_
mTAL	M	5.6	1.9	1.3	6.6
mTAL	F	2.3	0.5	1.2	4.4

Abbreviations: mTAL, medullary thick ascending limb; NHE3, sodium‐hydrogen exchanger 3; NKCC2, Na^+^/K^+^‐Cl^−^ cotransporter 2; PCT, proximal convoluted tubule; P_K_, K^+^ permeability.

In contrast, NBCe1 is essentially unrecoverable because it primarily belongs to the cell's acid–base (bicarbonate and pH) control system, not to the sodium–potassium balance that the measurements observe. From the data, one can defer how Na^+^ and K^+^ move and accumulate, so they tightly constrain the Na^+^/K^+^‐ATPase and K^+^ channels, and partly constrain NHE3 because it loads Na^+^ into the system. NBCe1, however, has the primary objective of moving bicarbonate, and changes in its activity can be compensated by buffering, CO_2_, Cl^−^ pathways, and other acid–base processes without altering Na^+^ or K^+^ levels or fluxes. As a result, many different NBCe1 values can produce the same Na^+^/K^+^ observables, making NBCe1 fundamentally unidentifiable from those measurements alone.

We perform the analogous computations with the mTAL models, in which we train the NNs using the same observables, and ask the models to infer NKCC2, Na^+^/K^+^‐ATPase activity, and apical and basolateral K^+^ permeabilities. MSE in model predictions, shown in Table 3, indicates good accuracy in all four transport parameters, as they all play significant roles in Na^+^ and K^+^ fluxes.

Taken together, these results underscore that parameter identifiability is not intrinsic to the NN framework, but depends on the choice of observable inputs and their physiological coupling to the parameters of interest.

## 4 DISCUSSION

This study demonstrates the feasibility and utility of NN–based surrogates for approximating solutions to complex models of renal epithelial transport. Conventional nonlinear solvers for these models are frequently hampered by convergence failures arising from the stiffness of the governing equations and the limited basin of attraction for initial guesses. In contrast, the NN‐based approach provides a robust and efficient alternative that circumvents these numerical limitations, thereby lowering technical barriers and making detailed epithelial transport modeling accessible to researchers without specialized expertise in numerical methods.

The computational pipeline that allows one to predict cellular variables involves the following steps:

1. Choose the cell model and decide on input and output variables.
  - The first step is for the user to choose a computational model of renal epithelial transport that represents the solutes and transporters that are of interest.
  - Then the user decides what the input features and output variables for the NN are. The latter is obvious—What would the user like the model to predict? Typical output variables include the cytosolic conditions and membrane potentials (used in this study) as well as transmembrane fluxes.
  - Input features are model parameters (e.g., expression level of a transporter) that the user intends to investigate over a range or to assign values that differ from the ones in the published version.
2. Generate training data. A substantial amount of data is typically required to train a NN. When such data are not already available, they can be generated by repeatedly running the epithelial transport model across a range of input parameter values.
3. Train and test the NN. The chosen input features and output variables will determine the architecture of the NN, for example, how many hidden layers and how many nodes for each layer. The NN is then trained and validated using data generated in Step 2.
4. Make model predictions. Once the NN is trained and validated, the user can make predictions for their specific set of parameters.

**FIGURE 4:**
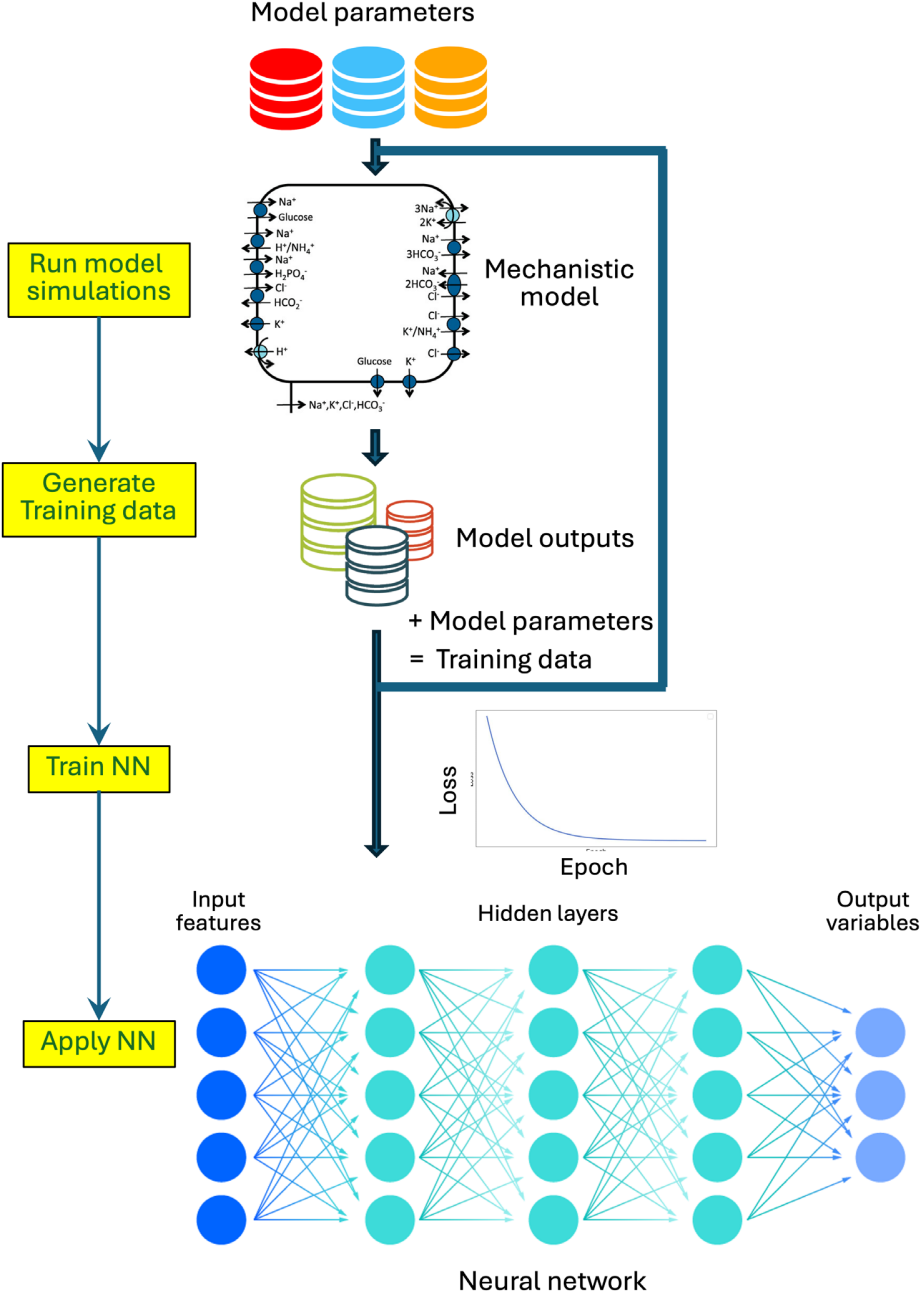
Workflow for training and applying a neural network (NN) surrogate of a mechanistic epithelial transport model. A mechanistic model is repeatedly simulated over a range of transport parameter combinations to generate steady‐state cellular outputs. The resulting model outputs, together with the corresponding transport parameters, form the training and validation datasets used to train a NN. Once trained, the NN serves as a fast surrogate that maps transport parameters to cellular variables and can be applied to previously unseen parameter sets. A schematic of a proximal tubule epithelial cell model is shown for illustration, but the workflow is applicable to other renal epithelial cell types. The NN architecture depicted includes three hidden layers for visualization purposes; in practice, the number of layers and nodes should be tailored to the dimensionality and complexity of the chosen inputs and outputs.

The workflow is illustrated in Figure 4.

It is important to clarify that not all steps in this workflow are intended to be performed by end users. In particular, generating training data (Step 2) and training the NN (Step 3) require computational expertise and are most naturally carried out by model developers or specialized groups. Once trained, however, the NN surrogate can be distributed and used independently, allowing users to evaluate model predictions for specified parameter values without engaging directly with the underlying numerical solvers. In this way, the framework separates model construction from model application and enables non‐computational researchers to interact with mechanistic models through a simplified and robust interface.

At present, this approach does not eliminate all barriers to adoption, and its practical impact will depend on the availability of shared, well‐documented surrogate models and user‐friendly tools. Nevertheless, by removing the need for solver initialization and reducing sensitivity to parameter changes, the NN surrogate provides a pathway toward broader accessibility of epithelial transport modeling.

Our results demonstrate that the trained NN models accurately predict key cellular variables, including cytosolic ion concentrations and membrane potentials. The proposed framework is readily extensible to incorporate (i) additional input variables, such as transporters not considered in the present study (e.g., SGLT2 in the PCT); (ii) additional outputs, including cell volume or other fluxes; and (iii) other renal epithelial cell types. Achieving high predictive accuracy requires that the selected input variables be sufficiently informative of the desired outputs, that the network architecture be appropriately specified, and that an adequate volume of training data be provided.

The NNs also perform well under transporter inhibition scenarios, including reduced NHE3 activity in the PCT and NKCC2 activity in the mTAL (Figure 2). The relatively low prediction errors observed for most variables indicate that the models can extrapolate beyond baseline transporter settings, an important capability for simulating pharmacological interventions, genetic perturbations, or pathological conditions.

In a complementary application, we show that the NN can infer selected membrane transport properties from observable cellular and flux data. This inverse modeling task is particularly relevant in experimental settings where direct measurement of transporter activity is challenging, but related quantities—such as solute concentrations, membrane potentials, or fluxes—are accessible. As discussed above, transport properties can be reliably inferred only when they are strong determinants of the observed variables, as illustrated by the larger errors for Na^+^–HCO_3_^−^ cotransporter activity (Figure 3d) compared with Na^+^/K^+^‐ATPase activity and basolateral K^+^ permeability (Figure 3a,b). Transport properties can also be incorporated as inputs; for example, in the PCT, cytosolic Na^+^ and K^+^ concentrations, basolateral membrane potential, and Na^+^/K^+^‐ATPase activity could be used to infer NHE3 activity and basolateral K^+^ permeability.

By eliminating the need for careful initialization and nonlinear solver tuning, this approach substantially lowers the barrier to using mechanistic epithelial transport models. Researchers can rapidly and reliably generate predictions across a wide range of physiological and pathophysiological conditions, facilitating broader adoption of computational tools for hypothesis generation and data interpretation. Potential applications include (i) predicting cytosolic composition, membrane potentials, and transmembrane or paracellular fluxes from measured transport properties and bath conditions; (ii) evaluating how cellular states and fluxes respond to perturbations in transport parameters or extracellular conditions; and (iii) inferring unmeasured transport properties from partial physiological observations.

An additional potential application of the NN surrogate is to provide initial guesses for traditional nonlinear solvers. In this hybrid approach, the NN prediction would serve as a fast approximation to the steady‐state solution, which could then be refined using a mechanistic solver. This strategy may be particularly valuable when exploring parameter regimes far from baseline conditions, where standard initialization methods often fail. By placing the solver closer to the basin of attraction of the true solution, the NN surrogate could improve convergence and expand the range of tractable simulations.

### 4.1 Limitations and future work

While promising, this approach has important limitations. As with any data‐driven method, the predictive accuracy of the NN ultimately depends on the quality, diversity, and coverage of the training data. Prediction errors are expected to increase when the model is applied to conditions that lie outside the distribution represented in the training set.

The present study is intentionally limited in scope, with a small number of input features and output variables chosen to demonstrate feasibility in a controlled setting. In contrast, full epithelial transport models may include many more parameters and state variables. Scaling the NN surrogate to higher‐dimensional input and output spaces is conceptually straightforward but requires larger and more diverse training datasets, as well as more expressive network architectures. Importantly, however, not all model parameters exert equal influence on a given set of outputs; in many applications, a subset of dominant transporters or pathways may be sufficient to capture the physiological behavior of interest. Thus, practical implementations of the NN framework may continue to rely on targeted feature selection rather than attempting to learn the full high‐dimensional mapping.

Even within the reduced setting considered here, prediction errors are non‐negligible for certain variables, particularly under extrapolative conditions such as strong transporter inhibition. These errors reflect both the limited coverage of the training data and the differing sensitivity of model outputs to specific transport processes. Expanding the training data to encompass a broader range of physiological and pharmacological conditions would likely improve robustness and predictive accuracy.

The current framework is formulated for an isolated epithelial cell. A natural and valuable extension is to model entire nephron segments by coupling multiple epithelial cell models in series and computing how luminal fluid composition evolves along the length of the segment. In such a multiscale setting, luminal fluid and solute transport can be described using mass conservation equations. Given appropriate boundary conditions at the segment inlet—such as inflow rate and solute concentrations—fluid and solute fluxes across individual epithelial cells can be computed sequentially. Using apical and paracellular fluxes predicted by the NN surrogate, luminal flow rate and solute concentrations can be updated step by step along the segment. Because the resulting conservation equations are typically well conditioned numerically, they can be solved efficiently using standard methods, enabling scalable nephron‐level simulations and, through coupling with vascular and interstitial compartments, providing a pathway toward whole‐kidney modeling. In these coupled systems, however, errors introduced at the level of individual cell predictions may propagate along the tubule. Nevertheless, because the governing luminal equations are well behaved and prediction errors for key variables are relatively small under baseline conditions (e.g., cytosolic solute concentrations and basolateral membrane potential; see Tables 1 and 2 and Figure 2), we do not expect error accumulation to be prohibitive. Nevertheless, quantifying error propagation in coupled epithelial systems remains an important direction for future work.

While the present study focuses on Na^+^ and K^+^, the underlying epithelial transport cell models represents 15 solutes and the corresponding transport parameters. It is straightforward to extend the NN model to include different or additional solutes (e.g., HCO_3_^−^) and parameters as features and output variables (e.g., basolateral or paracellular fluxes).

Looking forward, one potential extension is the development of a foundation model, defined here as a large NN trained on diverse datasets spanning multiple nephron segments, sexes, ages, species, and physiological conditions, which can then be fine‐tuned for specific cell types or experimental questions. Such a foundation model could capture shared transport principles across epithelial systems while retaining the flexibility to adapt to specialized contexts, thereby improving generalization and enabling broader reuse across renal physiology and pathophysiology. A foundation model would also be much more complex than the NN used in this study, with many more layers and nodes; therefore, requiring much more data to train.

## 5 CONCLUSION

In summary, this study presents a NN–based framework that fundamentally expands how renal epithelial transport models can be solved and used. By replacing fragile numerical solvers with a fast, stable surrogate learned from high‐fidelity simulations, the approach enables reliable model evaluation across a wide range of physiological conditions and transporter perturbations. This eliminates solver failures that have historically limited scalability, reproducibility, and broader use of epithelial transport models. By lowering technical barriers and enabling rapid, robust exploration of transport dynamics, the framework empowers experimental biologists to directly engage with quantitative modeling in their own systems. More broadly, this work opens new opportunities for integrating mechanistic transport models into experimental workflows, accelerating discovery and translation in nephrology.

## AUTHOR CONTRIBUTIONS

**Anita T. Layton:** Conceptualization; data curation; formal analysis; funding acquisition; investigation; methodology; project administration; resources; software; supervision; validation; visualization.

## Data Availability

Jupyter notebooks for this study are publicly available on GitHub: https://github.com/Layton‐Lab/Neural‐Network‐based‐Solver.
